# Overexpression of PSMC2 promotes the tumorigenesis and development of human breast cancer via regulating plasminogen activator urokinase (PLAU)

**DOI:** 10.1038/s41419-021-03960-w

**Published:** 2021-07-09

**Authors:** Yanyan Wang, Mingzhi Zhu, Jingruo Li, Youyi Xiong, Jing Wang, Haihong Jing, Yuanting Gu

**Affiliations:** Department of Breast Surgery, The First Affiliated Hospital of Zhengzhou University, Zhengzhou, Henan China

**Keywords:** Cancer, Breast cancer

## Abstract

Emerging evidence has declared that Proteasome 26S subunit ATPase 2 (PSMC2) is involved in tumor progression. However, its role in breast cancer has not been investigated. Therefore, we sought to establish a correlation between breast cancer and PSMC2. PSMC2 expression in tissues was detected by immunohistochemistry. Loss-of-function study was used to evaluate the effects of PSMC2 knockdown in cell proliferation, apoptosis and migration. A gene microarray was performed to explore the potential downstream of PSMC2 with the help of Ingenuity Pathway Analysis (IPA). The effects of the PSMC2/PLAU axis on breast cancer were examined in vitro. Compared to para-cancer tissues, PSMC2 level was considerably elevated in breast cancer, which was significantly correlated with tumor grade. Knockdown of PSMC2 suppressed breast cancer progression in vitro and in vivo. The mechanistic research revealed that PSMC2 promotes the development and progression of human breast cancer through interacting with PLAU. Outcomes of our study showed that overexpression of PSMC2 provide tumorigenic and metastatic advantages in breast cancer, which may involve the regulation of PLAU. This study not only reveals a critical mechanism of breast cancer development, but also provides a promising therapeutic target for breast cancer treatment.

## Introduction

Breast cancer (BC) is the most common cancer in women throughout the world and the second leading cause of cancer-related deaths after lung cancer [1, 2]. Although sophisticated diagnostics, accompanied with newer surgical techniques and radiation therapies, have reduced the recurrence rate and treatment-associated morbidity [3], treatment efficiency and prognosis of breast cancer patients are still not satisfactory because of the covert symptoms and late diagnose [4]. Therefore, the exploration of novel and more specific target for developing molecular targeted drug of breast cancer is of great significance and urgency.

26S Proteasome, which consists of the 20S core catalytic subunit and the 19S regulatory subunit, is extremely necessary for the quick destruction of key intracellular regulatory proteins, such as transcription factors and cell cycle regulators [5, 6]. Proteasome 26S subunit ATPase 2 (PSMC2) is a key member of the 19S regulatory subunit of 26S proteasome. Based on the close relationship between PSMC2 and the 26S proteasome, oncologists have launched a span-new theoretical branch concerning PSMC2 and malignancy. Nijhawan et al. first reported that downregulation of PSMC2 may suppress tumor growth of ovarian cancer [7]. Then Jing Qin et al. found that PSMC2 promoted the pancreatic cancer cell proliferation, apoptosis and migration [8]. Although PSMC2 was regarded as a novel synthetic lethal interaction relevant to human cancer, the functional validation and mechanism research for PSMC2 in BC is still unclear.

After screening the potential downstream of PSMC2, we identified plasminogen activator urokinase (PLAU) as a promising candidate. Unlike other urokinase, PLAU, encoding urokinase-type plasminogen activator (uPA), does not have kinase activity, but functions as a protease, and belongs to the PA family serine protease. It is mainly responsible for the transformation of plasminogen to plasmin, the hydrolysis of extracellular matrix remodeling related proteins and the activation of growth factors. Studies also indicated that PLAU can activate MAPK and JAK-STAT signaling pathways and focal adhesion kinase system directly or indirectly by binding with uPAR (urokinase plasminogen activator receptor), thus promoting ECM to release growth factors which are involved in proteolysis, cell migration, tissue remodeling, and cell adhesion. It has been found that PLAU is overexpressed in many types of human cancers [9–11], including breast cancer. It is interesting that, although Kozlova et al. found that PLAU was implicated in the inhibition of proliferation and motility of breast cancer cell lines [12], the overexpression of PLAU could increase the migration and invasion of breast cancer cells, which is related to the poor prognosis of breast cancer [13, 14]. Despite, the upstream driver of PLAU in regulating the development and progression of BC is still unclear.

Herein, we showed that PSMC2 is overexpressed in human breast cancer and is associated with advanced tumor grade. Using complementary molecular approaches in multiple cellular models in vivo and in vitro, we demonstrated that PSMC2 plays an important role in tumorigenesis and the development of breast cancer through the regulation of PLAU signaling. Consequently, as with PLAU, PSMC2 may be a viable therapeutic target in breast cancer.

## Materials and methods

### Clinical samples

The Ethics Committees of the First Affiliated Hospital of Zhengzhou University have approved all studies about human participants. All participants have written informed consents. 148 cases of breast cancer tissues and 12 cases of para-cancer normal tissues were obtained from the First Affiliated Hospital of Zhengzhou University and selected for this study. After surgical resection, all the breast cancer tissues and para-cancer normal tissues were frozen in the liquid nitrogen tank within 30 minutes.

### Mouse xenograft model

BALB/c nude mice (16–18 g, 4–5 weeks old, female) were purchased from Shanghai Jiesijie Experimental Animal Co., Ltd. (Shanghai, China). All mice were housed in a pathogen-free facility under a 12 h light-dark cycle with free access to food and water. The mouse experiments were approved by Ethics Committee of the First Affiliated Hospital of Zhengzhou University. 20 mice were randomly divided into two groups, shCtrl and shPSMC2. 10^6^ of shPSMC2 transfected MDA-MB-231 cells were injected subcutaneously. Tumors were evaluated by IVIS imaging system (emission wavelength of 510 nm) and quantified using IGOR Pro 4.09 A image analysis software. After sacrificed, tumors were measured by calipers and photographed, and volume was calculated.

### Breast cancer tissue microarray and immunohistochemistry (IHC)

Breast cancer tissue microarray (HBre-Duc150Sur-02, including 173 breast cancer samples and 35 para-carcinoma samples, purchased from Shanghai Outdo Biotech Company) was subjected to IHC analysis for PSMC2 protein expression. Briefly, the microarray was dehydrated with xylene and rehydrated with alcohol, then immersed into antigen-retrieval solution for antigen retrieval at 100 °C. After blocking with 3% H_2_O_2,_ the microarray was incubated with primary antibody overnight at 4 °C, then incubated with the second antibody for 2 h at room temperature (antibody information was shown in Table S1). DAB horseradish peroxidase color development Kit was used for visualizing and pictures were scanned by software of CaseViewer_2.0 and ImageScope_v11. Staining intensity was classified as 0: no staining, 1: light yellow, 2: brown yellow, 3: dark brown. The staining percentage scored as: 1: 1–24%, 2: 25–49%, 3: 50–74%, and 4: 75–100%. IHC scoring was determined by the staining intensity score and the staining percentage score.

### Ki-67 immunostaining

Mice tumor sections were fixed in 4% paraformaldehyde for 16 h. Paraffin embedded 5 μm sections were made for H&E and IHC staining. Citric acid buffer was added for antigen retrieval at 120 °C, then sections were blocked using PBS-H_2_O_2_ with 0.1% Tween 20. Ki67 antibody and secondary antibody (Table S1) were incubated. Color was developed with diaminobenzene and counterstained with hematoxylin. For H&E staining, Hematoxylin and Eosin were added for coloring. Stained slides were pictured with a microscope.

### Cell culture and transfection

The human mammary epithelial cell line HBL-100 and breast cancer cell lines MCF-7, MDA-MB-231 were obtained from the Cell Bank of Sciences (Shanghai, China). After resuscitation, the cells were cultured and stored according to the supplier’s instructions. The cells were maintained in Dulbecco’s modified Eagle’s Medium (DMEM) containing 10% FBS, 2 mM l-glutamine, 100 μg/ml streptomycin, and 100 U/ml penicillin at 37 °C and 5% CO_2_, and never passaged longer than 6 months and tested routinely by Hoechst DNA staining to ensure no mycoplasma contamination. For establishing stable transfection cells with PSMC2 knockdown and/or PLAU knockdown/overexpression, the cells were transfected with PSMC2 shRNA (shPSMC2) and/or PLAU shRNA (shPLAU) (vector: BR-V-108, sequences were listed in Table S2, Shanghai Yibeirui Biomedical Science and Technology Co., Ltd) or PLAU overexpression construct (PLAU group, Vector LV-007 as negative control, amplimer sequences were listed in Table S2, Shanghai Yibeirui Biomedical Science and Technology Co., Ltd), respectively. Stable clones were selected with blasticidin (2 μg/ml) and puromycin (300 ng/ml) for 4 weeks, respectively.

### Real-time quantitative PCR

Total RNA was extracted from the indicated cells by using Trizol according to the manufacturer’s instructions (Sigma, T9424-100m). Reverse transcription was performed with the Promega M-MLV Kit (M1701). Real-time PCR was performed using SYBR green PCR master mix (Life Technologies, Thermo Fisher Scientific). Primers used are listed in Table S3. For all RT-qPCR analysis, β-actin was used to normalize RNA input, and expression levels were calculated according to the comparative Ct method (ΔΔCT). The results were representative of at least three independent experiments.

### Western blot

Equal quantities of cellular proteins, prepared in sample lysis buffer and heated for 10 min at 100 °C, were electrophoresed through a 10% SDS/polyacrylamide gel and transferred to polyvinylidene fluoride membranes (PVDF, Millipore). The membranes were incubated with special first and secondary antibodies which detailed in Table S1. Signals were detected using a CL-PLUS Kit (Amersham) and films were analyzed with ImageJ software.

### Co-immunoprecipitation

For immunoprecipitation, agarose beads coupled with PSMC2-antibody was prepared and mixed with cell lysates, which harvested from MDA-MB-231 breast cancer cells, and rotated at 4 °C for 1 h. Immuno-complexes separated from agarose beads were washed with lysis buffer and then suspended with SDS blue loading buffer. After the beads were boiled, the precipitated proteins were separated by SDS-PAGE and transferred to PVDF membranes for further analysis. WB analysis was used to detect proteins.

### 3-(4,5-Dimethyl-2-thiazolyl)-2,5-diphenyl-2H-tetrazolium bromide (MTT) assay

Cells were seeded in 96-well plates at 3,000 cells/well in triplicate. At each time point, cells were stained with 100 μl of sterile MTT dye (0.5 mg/ml, Sigma) for 4 h at 37 °C, followed by removal of the culture medium and addition of 150 μl of dimethyl sulfoxide (Sigma). After treated with dimethyl sulfoxide, the absorbance was measured at 490 nm with a spectrophotometric plate reader and cell viability was calculated.

### Apoptotic rate analysis by flow cytometry

The cells with different treatments were plated in 6-well plates. After 48 h incubation, cells were harvested and washed by 1× PBS twice and then for apoptosis analysis by flow cytometry (Guava easyCyte HT). The cells were resuspended by 500 µl binding buffer, stained by Annexin V-FITC according to the Annexin V-FITC apoptosis detection kit (eBioscience, 88-8007-74) and detected by flow cytometry.

### Cell cycle detection by flow cytometry

Ice-cold 70.0% alcohol was used to fix and permeabilize cells at 4 °C overnight. Washed with phosphate buffer, cells were dealt with RNase for 20 min at 37 °C and then stained with 50 μg/ml propidium iodide (PI, Sigma, P4170). FACSCalibur flow cytometer (BD) was used to analyze DNA content of cells. For each sample, a total of 10,000 events were counted. The percentage of cells with different phase (G1, S and G2/M) was analyzed and determined by CellQuest software (BD).

### Colony formation assay

Cells were plated at 500 cells per well in six-well plates in triplicate and cultured for 14 days. Colonies were fixed with 10% formaldehyde for 15 min, stained with 0.5% crystal violet for another 15 min, and counted under an inverted microscope.

### Wound-healing assay

Cell migration ability was measured using the Wound-Healing Assay. Briefly, cells were seeded on six-well plates with DMEM containing no FBS and grown to monolayer confluence. The monolayers were scratched with a sterile pipette tip to create straight wounds and then incubated with DMEM containing 10% FBS. At 0 and 24 h after wounding, images were captured and migration was documented using an inverted Olympus IX50 microscope.

### Transwell Assay

Cells (1 × 10^4^) were plated on the top side of polycarbonate Transwell filters coated with Matrigel in the upper chamber of BioCoat^TM^ Invasion Chambers (BD Biosciences) and incubated at 37 °C for 24 h, followed by removal of cells inside the upper chamber using cotton swabs. Migrated cells on the lower membrane surface were fixed in 1% paraformaldehyde, stained with hematoxylin, and counted (ten random fields per well, ×100 magnification).

### Celigo cell counting assay

Lentivirus-infected MDA-MB-231 cells were seeded into a 96-well plate with 3000 cells per well for culturing. The plate was continuously detected by Celigo (Nexcelom) for 5 days at the same time. The number of survival cells was counted and the cell proliferation rate was analyzed.

### Human apoptosis antibody array

Human Apoptosis Antibody Array (R&D Systems) was performed in MCF-7 cells transfected with shPSMC2 or shCtrl following the manufacturer’s instructions. Briefly, protein samples were extracted and diluted with Array Diluent Buffer. Each array membrane was blocked and then incubated with protein samples (0.5 mg/mL) overnight at 4 °C. After washing, Arrays were incubated with the primary antibody for 2 h, following incubated with HRP linked Streptavidin secondary antibody. Positive signals were captured by ECL plus kit and the signal densities were analyzed with software ImageJ (National Institute of Health).

### Human GeneChip primeview analyzing

Total RNA from shPSMC2 knockdown MCF-7 and related control cells were collected for Human GeneChip primeview analyzing according to the manufacturer’s guidelines. Briefly, RNA was prepared from 100 ng total RNA using the 3’IVT Express Kit, then hybridized on a GeneChip Human array at 45 °C. Next, the array was washed and stained by Affymetrix Fluidics Station 450, and scanned by the Affymetrix GeneChip Scanner. The data processing and analysis were performed with R studio. Limma package was used for cluster analysis and differential expression of genes assessing. Canonical pathways, diseases and functions, molecular and cellular processes that are significantly associated with differentially expressed genes (DEGs) in the data sets were determined using Ingenuity Pathway Analysis (IPA) software. |*Z* score | > 2 is significant.

### Statistical analyses

Graphs were made using GraphPad Prism 8.0 (GraphPad Software) and all statistical analysis was performed using SPSS 17.0 (IBM) and *P* < 0.05 was considered statistically significant. Data are expressed as the mean ± SD, Student’s *t* test or one-way ANOVA was used to analyze the statistical significance. PSMC2 gene expression difference revealed in IHC analysis was analyzed by Rank Sum test, and Mann–Whitney U analysis and Spearman Rank correlation analysis was used to analyze the relationship between PSMC2 expression and patients’ tumor characteristics.

## Result

### High expression of PSMC2 is associated with aggressive phenotypes in human breast cancer

**Fig. 1:**
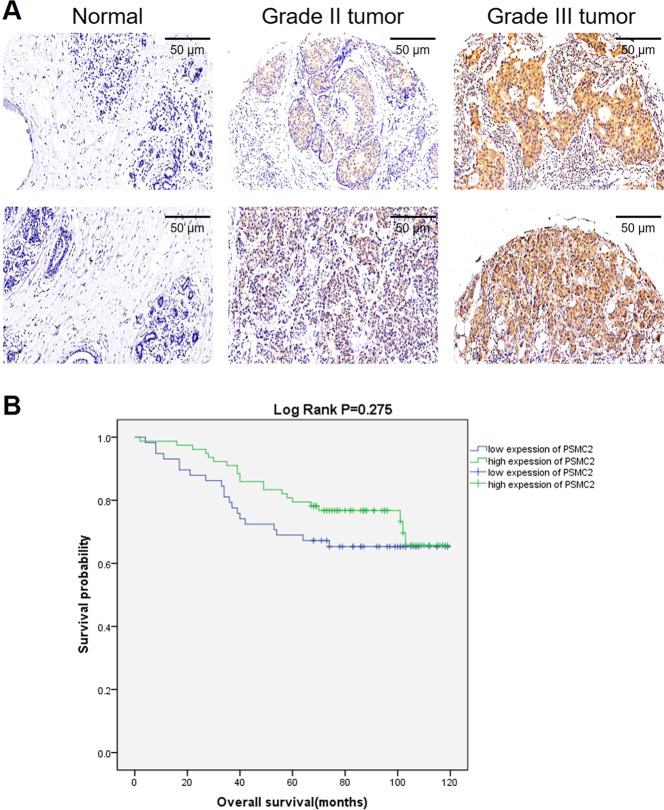
PSMC2 is upregulated in breast cancer and predict poor prognosis. **A** The expression of PSMC2 in breast cancer tissues and normal tissues was detected by IHC. **B** Kaplan–Meier survival analysis was used to establish the relationship between PSMC2 expression and survival period of breast cancer patients.

In order to investigate the PSMC2 expression level in breast cancer specimens, a breast cancer tissue microarray consisting of 173 breast cancer samples and 35 para-carcinoma samples was used for IHC staining. Based on the IHC score median of PSMC2 in all samples, high PSMC2 expression was observed in 53.2% breast cancer tumor samples and in no para-carcinoma tissues, which was indicated in Table 1. Fig. 1A shows the representative images of tissue microarrays. Moreover, we analyzed the relationship between PSMC2 expression level and tumor characteristics in patients with breast cancer (Table 2). In this study, we found that PSMC2 expression possesses a significant relationship with the grade and age of breast cancer. Notably, PSMC2 expression presented a positive correlation with the pathological grade of breast cancer (Table 2 and S4). Moreover, Kaplan–Meier survival analysis was performed, indicating the positive correlation between high PSMC2 expression and poorer prognosis (Fig. 1B). In summary, these results revealed that high levels of PSMC2 expression might be related to breast cancer development and may serve as a prognostic biomarker.

**Table 1:** Expression patterns of PSMC2 in breast cancer tissues and normal tissues revealed in immunohistochemistry analysis.

PSMC2 expression	Tumor tissue		Normal tissue	
	Cases	Percentage	Cases	Percentage
Low	81	46.8%	35	100%
High	92	53.2%	0	–

*P* < 0.001

**Table 2:** Relationship between PSMC2 expression and tumor characteristics in patients with breast cancer.

Features	No. of patients	PSMC2 expression		*P* value
		Low	High	
All patients	175	83	92	
Age (years)				0.072
≤60	108	56	52	
>60	59	22	37	
Tumor size				0.020
≤3 cm	87	45	42	
>3 cm	59	19	40	
Grade				0.006
I	1	0	1	
II	81	45	36	
III	67	21	46	
T Infiltrate				0.347
T1	42	22	20	
T2	100	45	55	
T3	14	5	9	
T4	1	1	0	
Lymphatic metastasis (N)				0.238
N0	85	37	48	
N1	42	19	23	
N2	21	12	9	
N3	12	7	5	
AJCC Stage				0.637
1	30	15	15	
2	88	37	51	
3	39	21	18	
Lymphoid positive number				0.475
=0	78	32	46	
>0	66	31	35	

### Silencing of PSMC2 inhibits cell growth, enhances apoptosis and decreases the migration capability in vitro

**Fig. 2:**
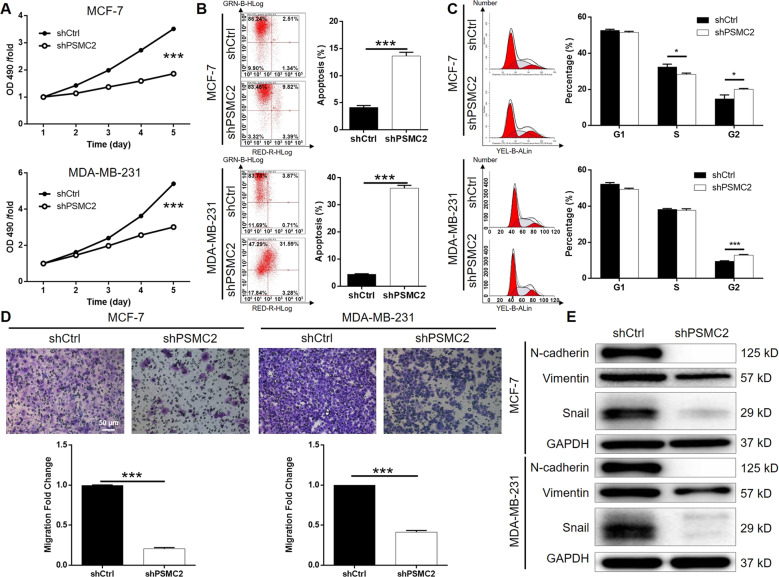
Silencing of PSMC2 inhibits breast cancer development in vitro. **A** MTT assay showed that knocking down the expression of PSMC2 in breast cancer cells inhibited cell proliferation. **B** Flow cytometry suggested that PSMC2 knockdown promoted apoptosis of breast cancer cells. **C** The detection of cell cycle distribution indicated that PSMC2 knockdown induces cell cycle arrest in G2 phase. **D** Transwell assay displayed the weakened migration ability of breast cancer cells after silencing PSMC2. **E** The expression of EMT markers including N-cadherin, Snail and Vimentin was decreased in PSMC2 knockdown cells. Data were shown as mean ± SD (*n* ≥ 3). * *P* < 0.05, *** *P* < 0.001.

To determine the importance of PSMC2 in breast cancer development, we silenced PSMC2 *via* lentivirus-mediated transferring of short hairpin RNAs (shRNAs) into two human breast cancer cell lines (MCF-7, MDA-MB-231), which expressed significantly higher PSMC2 than mammary epithelial cell line HBL-100. MCF-7 and MDA-MB-231 cells were infected with shCtrl or shPSMC2 in an efficiency of >80% (Fig. S2). qRT-PCR verified the significant knockdown of PSMC2, and western blot analysis demonstrated the same results in protein level. Next, we evaluated the cell proliferation with 3-(4,5-dimethyl-2-thiazolyl)−2,5-diphenyl tetrazolium bromide (MTT). The results showed that cells transfected with shPSMC2 grew significantly slower than the shCtrl cells (Fig. 2A). To further verify this issue, we used flow cytometry to analyze cell apoptosis and cell cycle distribution in PSMC2 silenced breast cancer cells. The results revealed that lower expression of PSMC2 in breast cancer cells markedly induced apoptosis compared with shCtrl cells (Fig. 2B). PSMC2 depletion in MCF-7 cells leads to a reduced cells population in S phase whereas a significant arrest in G2 (Fig. 2C). Similarly, enhanced G2 phase arrest was also determined in PSMC2 silenced MDA-MB-231 cells. What’s more, migration capability are important characteristics of malignant tumors. Significantly reduced migration ability was observed in MCF-7 and MDA-MB-231 cells upon PSMC2 knockdown determined by a transwell based assay (Fig. 2D), which may be explained by the inhibited epithelial-to-mesenchymal transition (EMT) represented by the downregulation of N-cadherin, Vimentin and Snail (Fig. 2E).

To identify the mechanism of PSMC2 in apoptosis regulation, we performed an unbiased screen on a human apoptosis antibody array, which measured the expression of 43 apoptosis-related proteins in shCtrl or shPSMC2 cells. Compared with the shCtrl group, the expression of Caspase8 and CD40 was higher in the shPSMC2 group, but the expression of cIAP-2, HSP27, IGF-1sR, Livin, Survivin, TRAILR-3, TRAILR-4, and XIAP was lower in the shPSMC2 group (Fig. S3).

### Silencing of PSMC2 inhibits tumorigenicity of breast cancer cells in vivo

**Fig. 3:**
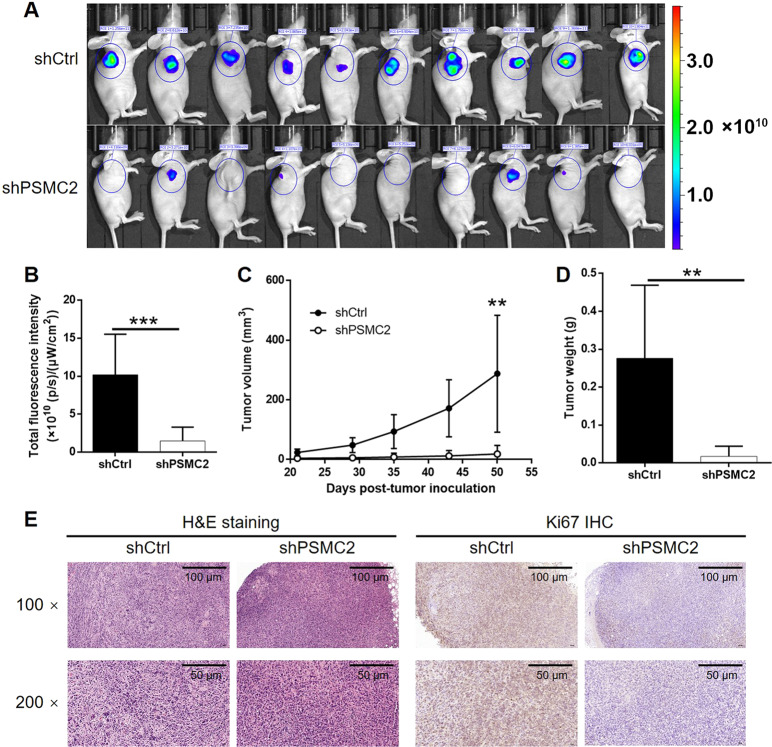
Knockdown of PSMC2 inhibits tumorigenesis and tumor growth of breast cancer in vivo. **A** In vivo imaging was performed to observe the tumor forming and growth in vivo. **B** The fluorescence intensity of tumor detected in animal imaging was scanned and quantified as a representation of tumor burden. **C** The measurement and calculation of tumor volume were carried out throughout animal culturing and showed that tumors formed by PSMC2 knockdown cells grew much slower. **D** The tumors formed by PSMC2 knockdown cells were significantly lighter than that formed by shCtrl cells. **E** The histological characteristics and Ki67 expression of the tumors formed in shPSMC2 or shCtrl group were examined by H&E staining and IHC, respectively. Data were shown as mean ± SD (*n* ≥ 3). * *P* < 0.05, * *P* < 0.01, *** *P* < 0.001.

To further determine the role of PSMC2 in breast cancer in vivo, animal models were constructed by subcutaneous injection of shCtrl or shPSMC2 breast cancer cells. We evaluated the tumor formation and the growth of established tumors and fluorescence *via* in vivo imaging (Fig. 3A). 5 weeks after injection, we observed much stronger fluorescence signals in the control group than the PSCM2-depleted group, and the difference was statistically significant (Fig. 3B). The measurement of tumor size as well as the tumor volume showed similar results that xenografts in the shPSMC2 group grew much slower than that in the shCtrl group (Fig. 3C). Subsequently, tumors were excised and then subjected to weighting after the mice were sacrificed. The averaged tumor weight of the shCtrl mice was significantly heavier than that of the silencing PSMC2 group (Fig. 3D). Besides, the H&E staining was used to authenticate the xenografts and the results of IHC staining showed that the expression of Ki-67 in shPSMC2 xenografts was lower than that in the shPSMC2 group (Fig. 3E, S4). These results clearly indicated that silencing PSMC2 expression inhibited breast cancer tumorigenicity in vivo.

### Silencing of PSMC2 alters PLAU expression in breast cancer cells

**Fig. 4:**
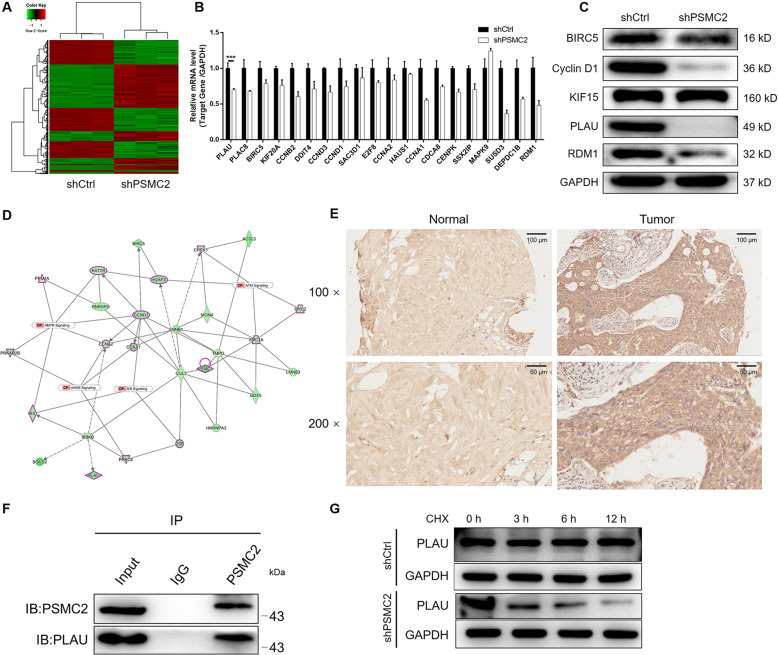
PSMC2 knockdown inhibits breast cancer through interacting and regulating PLAU. **A** The heatmap showed the differentially expressed genes identified by gene microarray analysis of shPSMC2 and shCtrl cells. **B**, **C** Several differentially expressed genes were selected for further verification by qPCR (**B**) and western blotting (**C**) based on the criteria of canonical signaling pathway enrichment analysis. **D** A molecular interaction network involving PSMC2 and the selected differentially expressed genes was constructed based on IPA. **E** IHC was performed to detect the expression of PLAU in normal tissues and breast cancer tissues. **F** Co-immunoprecipitation assay was performed to verify the interaction between PSMC2 and PLAU. **G** Upon the treatment of Cycloheximide (CHX, 50 μg/ml), the protein stability of PLAU in shCtrl or shPSMC2 cells was evaluated by western blotting. Data were shown as mean ± SD (*n* ≥ 3). *** *P* < 0.001.

To further evaluate the molecular mechanism of PSMC2 in regulating breast cancer, whole-genome Affymetrix GeneChip hybridization was adopted to discover PSMC2 silencing affected gene expression pattern and analyze the potential regulation manners. The screening criteria of significant difference genes were: |fold change | ≥ 1.6 and FDR < 0.05. There are 1700 genes that reached to the significant difference whose expression are affected by PSMC2 knockdown, of which 887 genes were upregulated and 813 genes were downregulated (Fig. 4A, S5A). In order to conduct further bioinformatics analysis of these differentially expressed genes (DEGs), enrichment analysis was performed based database of Ingenuity Pathway Analysis (IPA). We found that the expression of DEGs was mainly concentrated in classic pathways such as Superpathway of Cholesterol Biosynthesis, Cholesterol Biosynthesis I, eNOS signaling pathways (Fig. S5B). Otherwise, cancer was found to be the disease most enriched by the DEGs (Fig. S5C). Based on the above results, several DEGs were selected for qPCR and western blot verification, which showed the markedly downregulation of PLAU in PSMC2 knockdown cells, and for constructing PSMC2-centered interaction network (Fig. 4B–D). IHC analysis using anti-PLAU antibody in clinical specimens also visualized the upregulation of PLAU in breast cancer (Fig. 4E). Given the link between PSMC2 and PLAU, we next determined the mechanism of PLAU upregulation by PSMC2 in breast cancer. A co-immunoprecipitation assay was performed. Following immunoprecipitation of PSMC2, the existence of PLAU was detected in the complex (Fig. 4F), validating their interaction. Additionally, further investigation showed that knockdown of PSMC2 enhances the expression of ubiquitinated proteins and ubiquitinated PLAU, by which PSMC2 may regulates the protein stability and thus the expression of PLAU (Fig. 4G and S5D). Collectively, we deduced that PSMC2 may regulate breast cancer through PLAU.

### PLAU plays a crucial role in the PSMC2-induced regulation of breast cancer

**Fig. 5:**
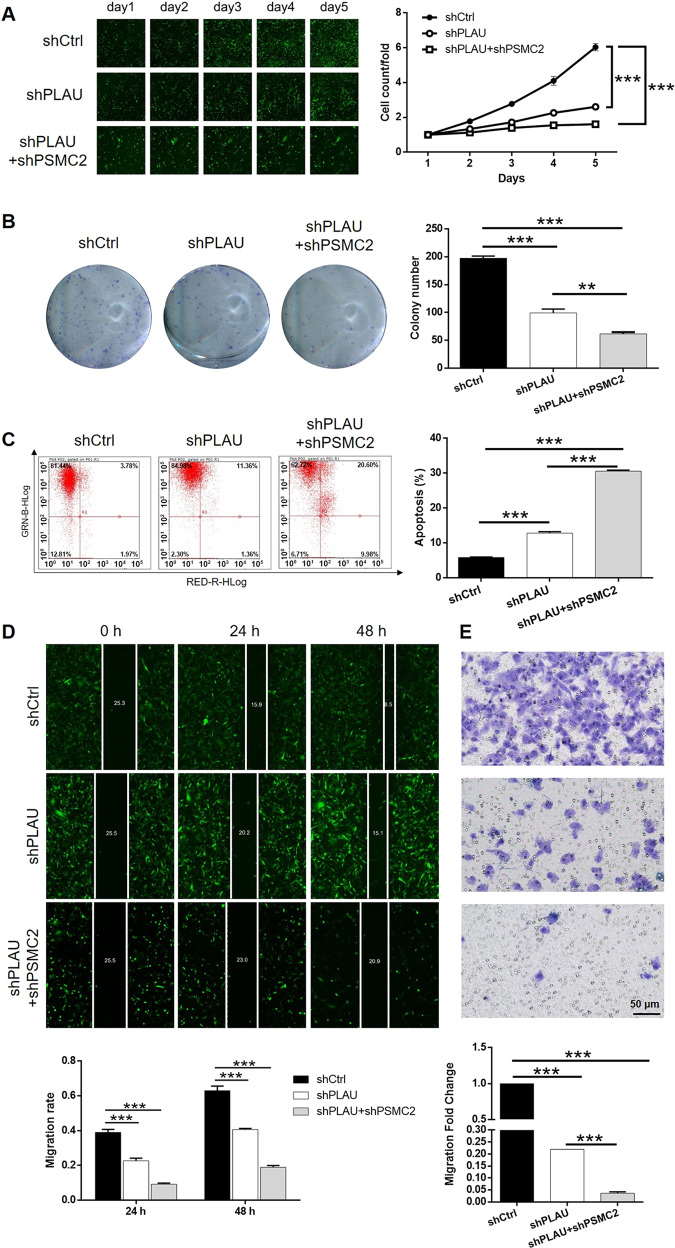
Knockdown of PLAU potentiates the inhibition of breast cancer induced by PSMC2 knockdown. **A** The effects of shPLAU or shPSMC2+shPLAU on cell proliferation were evaluated by celigo cell counting assay. **B** Colony formation assay was used to evaluate the effects of shPLAU or shPSMC2+shPLAU on colony formation ability of breast cancer cells. **(C)** The influence of shPLAU or shPSMC2+shPLAU on cell apoptosis was examined by flow cytometry. **D**, **E** The impact of shPLAU or shPSMC2+shPLAU on cell migration was determined by wound-healing assay (**D**) and transwell assay (**E**), respectively. Data were shown as mean ± SD (*n* ≥ 3). ** *P* < 0.01, *** *P* < 0.001.

**Fig. 6:**
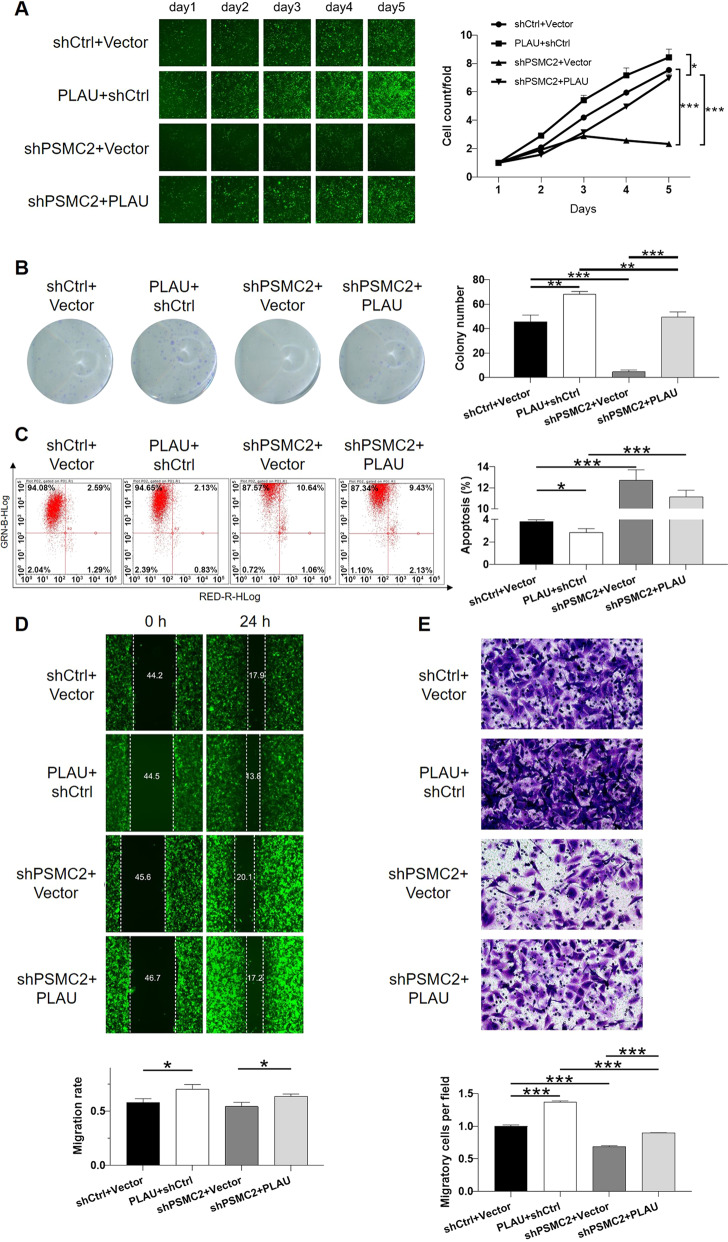
Overexpression of PLAU reverses the inhibition of breast cancer induced by PSMC2 knockdown. **A** The effects of mere PLAU overexpression, mere PSMC2 knockdown and simultaneous PLAU overexpression + PSMC2 knockdown on cell proliferation were evaluated by celigo cell counting assay. **B** Colony formation assay was used to evaluate the effects of mere PLAU overexpression, mere PSMC2 knockdown and simultaneous PLAU overexpression + PSMC2 knockdown on colony formation ability of breast cancer cells. **C** The influence of mere PLAU overexpression, mere PSMC2 knockdown and simultaneous PLAU overexpression + PSMC2 knockdown on cell apoptosis was examined by flow cytometry. **D**, **E** The impact of mere PLAU overexpression, mere PSMC2 knockdown and simultaneous PLAU overexpression + PSMC2 knockdown on cell migration was determined by wound-healing assay (**D**) and transwell assay (**E**), respectively. Data were shown as mean ± SD (*n* ≥ 3). * *P* < 0.05, ** *P* < 0.01, *** *P* < 0.001.

For the sake of further exploring whether PLAU mediated the functions of PSMC2 in breast cancer, lentivirus expressing shPLAU was prepared for infecting MDA-MB-231 cells alone or together with shPSMC2. Same as abovementioned, transfection efficiency and validity of gene knockdown were assessed by fluorescence imaging (Fig. S6), qPCR (Fig. S7A) and western blot (Fig. S7B), respectively. It is worth noting that mutual regulation between PLAU and PSMC2 could be observed in the results of western blot analysis (Fig. S7B). Subsequently, the cell phenotype detection showed that PLAU knockdown slowed down cell proliferation (Fig. 5A) and colony formation (Fig. 5B), elevated cell apoptosis rate (Fig. 5C) and suppressed cell migration ability (Fig. 5D for wound-healing assay and Fig. 5E for transwell assay). Moreover, the co-infection of shPLAU and shPSMC2 potentiate the inhibition effects of both PLAU and PSMC2 knockdown. More importantly, cell lines with mere PSMC2 knockdown (shPSMC2+Vector), mere PLAU overexpression (PLAU + shCtrl), or simultaneous PSMC2 knockdown and PLAU overexpression (shPSMC2+PLAU) were constructed for further demonstrating the synergistic effects of PSMC2 and PLAU on breast cancer development (Fig. S8). As shown in Fig. 6, the results not only illustrated that PLAU overexpression could promote the development of breast cancer through accelerating cell proliferation and colony formation, suppressing cell apoptosis and inhibiting cell migration, but also showed that PLAU overexpression could partially reverse the inhibitory effects of PSMC2 knockdown on breast cancer development. Therefore, herein, the cooperative effects of PLAU and PSMC2 on the development and progression of breast cancer were illustrated.

## Discussion

Breast cancer, a solid tumor characterized by high heterogeneity, has become a great threat to the health of contemporary women. Although various endocrine therapy drugs, chemotherapy drugs, molecular targeted drugs, and immunotherapy drugs have improved the prognosis of BC patients to some extent, the 5-year-survival rate and prognosis of patients are still unsatisfactory [15]. Therefore, the exploration and discovery of novel therapeutic targets with better specificity and efficiency are of great significance for treating breast cancer and improving the survival rate of BC patients to a greater extent. Previous work has proved that PSMC2 itself or as the downstream target of miR-630 could regulate osteosarcoma cells proliferation, apoptosis and migration [16, 17]. A similar function of PSMC2 as tumor promotor was also observed in pancreatic cancer and colorectal cancer [18, 19]. However, till now, the biological function of PSMC2 in human breast cancer and the underlying mechanism were still unclear.

The results of our study verified the high expression of PSMC2 in human breast cancer and the promotion effects of PSMC2 on its development and progression. Through the detection of tissue microarray, we found the overexpression of PSMC2 breast cancer. Besides, through statistical analysis, we also indicated that abnormal expression of PSMC2 might be related to aggressive tumor features, such as physiological grade. Moreover, we investigated the functions of PSMC2 in breast cancer cell proliferation, apoptosis, colony formation, migration, invasion and tumorigenesis via loss-of-functional studies. Tumor cells usually showed up the capacities of infinite proliferation, rare apoptosis and rapid colony forming. Our results indicated that breast cancer cells with comparatively low PSMC2 expression showed slower proliferation rate and weaker colony formation ability, with relatively higher apoptosis rate. Moreover, knockdown of PSMC2 also was able to inhibit the motility of breast cancer cells, suggesting its role in tumor metastasis, which is also an important property of malignancy. More importantly, the in vivo study using mice xenograft model strongly supported the critical role of PSMC2 expression in breast cancer development. Taken together, PSMC2 could potently facilitate tumorigenesis and metastasis in many respects throughout the progression of breast cancer.

To investigate the molecular mechanism of PSMC2 in breast cancer, an Affymetrix GeneChip analysis was performed to explore the striking changes of cancer-related genes among breast cancer cells with or without PSMC2 knockdown. Through IPA analysis, we found the downstream molecules and signaling pathways related to PSMC2. We recognized a positive correlation between PSMC2 and PLAU, which plays a crucial role in the metastatic process of breast cancer [20, 21]. *PLAU* is located on human chromosome 10q22.2 and encodes urokinase-type plasminogen activator (uPA). PLAU is a serine proteolytic enzyme widely present in animals. It is synthesized by monocytes, fibroblasts, epithelial cells and cancer cells, and is secreted by cells in the form of pro-uPA/sc-uPA [22–25]. Studies have shown that PLAU is closely related to tumor diagnosis, therapeutic targets and patient prognosis [26]. A state-of-art work indicated that PLAU, which is overexpressed in tumor tissues, functioned collaboratively with FOXM1 in the promotion of gastric cancer progression [27]. Suppression of PLAU by miR-193a-3p was also identified as a mechanism that inhibits the development and progression of colorectal cancer [9]. Moreover, PLAU was also reported to be involved in the pharmacology of triptolide to alleviate proliferation and migration of pancreatic cancer cells [28]. The correlation between PLAU and breast cancer has also been studied [21, 29]. Duffy *et al*. reported in the 1980s that uPA activity in primary breast cancer was related to tumor size, thus predicting that uPA may be a prognostic marker for breast cancer [30]. Janicke *et al*. detected high expression of uPA protein in breast cancer tissues by ELISA and high expression of uPA antigen in primary breast cancer tissues, and inferred that PLAU is related to the poor prognosis of breast cancer patients [31]. In our study, through further verification *via* co-IP, we confirmed that PSMC2 may interact with PLAU directly. Silencing PSMC2 leads the decrease in the protein stability and expression of PLAU, which may be related to the enhanced ubiquitination of PLAU upon PSMC2 knockdown. Knockdown or overexpression of PLAU could enhance or alleviate the inhibition of PSMC2 depletion on breast cancer development. Considering that an effect could be seen on RNA level, we are still working on the mechanism on RNA level by which PSMC2 regulates PLAU. Based on these results, we surmise that PSMC2 may regulate PLAU both on RNA level and protein level through different mechanism.

Our results showed that PSMC2/PLAU axis promoted the tumorigenesis and development of breast cancer. Based on these findings, decreasing the PLAU expression by inhibiting PSMC2 expression may provide new entry points for improving BRCA therapy.

## Supplementary information

Table S1

Table S2

Table S3

Table S4

Supplementary figure legends

Figure S1

Figure S2

Figure S3

Figure S4

Figure S5

Figure S6

Figure S7

Figure S8

## Data Availability

All data generated or analyzed during this study are included in this published article and its supplementary information files.
