# Supplementary material for: Overexpression of PSMC2 promotes the tumorigenesis and development of human breast cancer via regulating plasminogen activator urokinase (PLAU)
Source: Cell Death Dis. 2021 Jul 9;12(7):690. doi: 10.1038/s41419-021-03960-w (PMC8271021; doi:10.1038/s41419-021-03960-w)
Supplement: Supplementary file 1 — Table S1 [file 41419_2021_3960_MOESM1_ESM.docx]

Table S1 Antibodies used in western blotting and IHC

| Primary antibodies | Dilution in WB | Source species | Company | Catalog No. |
| --- | --- | --- | --- | --- |
| PSMC2 | 1:1000 | Mouse | Santa Cruz | SC-166972 |
| BIRC5 | 1:1000 | Rabbit | abcam | ab469 |
| Cyclin D1 | 1:2000 | Rabbit | CST | 2978 |
| KIF15 | 1:2000 | Rabbit | fine test | FNab04551 |
| PLAU | 1:1000 | Rabbit | abcam | ab133563 |
| RDM1 | 1:1000 | Rabbit | biorbyt | orb352658 |
| GAPDH | 1:3000 | Rabbit | Bioworld | AP0063 |
| N-cadherin | 1:1000 | Rabbit | abcam | ab18203 |
| Vimentin | 1:1000 | Rabbit | abcam | ab92547 |
| Snail | 1:1000 | Rabbit | CST | 3879S |
| Ubiquitin | 1:750 | Rabbit | Proteintech | 10201-2-AP |
| Primary antibodies | Dilution in IHC | Source species | Company | Catalog No. |
| PLAU | 1:150 | Rabbit | abcam | ab133563 |
| PSMC2 | 1:50 | Mouse | Santa Cruz | SC-166972 |
| Ki67 | 1:200 | Rabbit | abcam | ab16667 |
|  |  |  |  |  |
|  |  |  |  |  |
| Secondary antibody | Dilution |  | Company | Catalog No. |
| HRP Goat Anti-Rabbit IgG (WB) | 1:3000 |  | Beyotime | A0208 |
| HRP Goat Anti-Mouse IgG (WB) | 1:3000 |  | Beyotime | A0216 |
| HRP Goat Anti-Rabbit IgG (IHC) | 1:200 |  | Abcam | Ab111909 |
