# Supplementary material for: Overexpression of PSMC2 promotes the tumorigenesis and development of human breast cancer via regulating plasminogen activator urokinase (PLAU)
Source: Cell Death Dis. 2021 Jul 9;12(7):690. doi: 10.1038/s41419-021-03960-w (PMC8271021; doi:10.1038/s41419-021-03960-w)
Supplement: Supplementary file 2 — Table S2 [file 41419_2021_3960_MOESM2_ESM.docx]

Table S2 The target sequences and shRNA sequences

| Gene | No. | Target sequence (5'-3') | shRNA sequences (5'-3') | |
| --- | --- | --- | --- | --- |
| PSMC2 | Pbr-00145-a | GCCAGGGAGATTGGATAGAAA | CcggGCCAGGGAGATTGGATAGAAATTCAAGAGATTTCTATCCAATCTCCCTGGCTTTTTg | |
| PSMC2 | Pbr-00145-b | GCCAGGGAGATTGGATAGAAA | aattcaaaaaGCCAGGGAGATTGGATAGAAATTCAAGAGATTTCTATCCAATCTCCCTGGC | |
| PSMC2 | Pbr-23881-a | CAACGTAAAGCAGTTTGCCAA | CcggCAACGTAAAGCAGTTTGCCAActcgagTTGGCAAACTGCTTTACGTTGTTTTTg | |
| PSMC2 | Pbr-23881-b | CAACGTAAAGCAGTTTGCCAA | aattcaaaaaCAACGTAAAGCAGTTTGCCAActcgagTTGGCAAACTGCTTTACGTTG | |
| PSMC2 | Pbr-23882-a | AAGCAAGTTGAAGATGACATT | CcggAAGCAAGTTGAAGATGACATTctcgagAATGTCATCTTCAACTTGCTTTTTTTg | |
| PSMC2 | Pbr-23882-b | AAGCAAGTTGAAGATGACATT | aattcaaaaaAAGCAAGTTGAAGATGACATTctcgagAATGTCATCTTCAACTTGCTT | |
| PLAU | Pbr10246 | TTACCCAAAGAAGGAGGACTA | CCGGTTACCCAAAGAAGGAGGACTACTCGAGTAGTCCTCCTTCTTTGGGTAATTTTTG | |
| PLAU | Pbr10246 | TTACCCAAAGAAGGAGGACTA | AATTCAAAAATTACCCAAAGAAGGAGGACTACTCGAGTAGTCCTCCTTCTTTGGGTAA | |
| PLAU | Pbr10247 | CGGCTCTGAAGTCACCACCAA | CCGGCGGCTCTGAAGTCACCACCAACTCGAGTTGGTGGTGACTTCAGAGCCGTTTTTG | |
| PLAU | Pbr10247 | CGGCTCTGAAGTCACCACCAA | AATTCAAAAACGGCTCTGAAGTCACCACCAACTCGAGTTGGTGGTGACTTCAGAGCCG | |
| PLAU | Pbr10248 | GTGGATGTGCCCTGAAGGACA | CCGGGTGGATGTGCCCTGAAGGACACTCGAGTGTCCTTCAGGGCACATCCACTTTTTG | |
| PLAU | Pbr10248 | GTGGATGTGCCCTGAAGGACA | AATTCAAAAAGTGGATGTGCCCTGAAGGACACTCGAGTGTCCTTCAGGGCACATCCAC | |
| Amplimer sequences for PLAU overexpression (5'-3') | | | | |
| A0127 (PLAU)-F | | | | GATTCTAGAGCTAGCGAATTCCGCCACCATGGTCTTCCATTTGAGAACTAG |
| A0127 (PLAU)-R | | | | TCCTTCGCGGCCGCGGATCCTCAGAGGGCCAGGCCATTCTCTTC |
|  | | | | |
