# Supplementary material for: Overexpression of PSMC2 promotes the tumorigenesis and development of human breast cancer via regulating plasminogen activator urokinase (PLAU)
Source: Cell Death Dis. 2021 Jul 9;12(7):690. doi: 10.1038/s41419-021-03960-w (PMC8271021; doi:10.1038/s41419-021-03960-w)
Supplement: Supplementary file 3 — Table S3 [file 41419_2021_3960_MOESM3_ESM.docx]

Table S3 Primers used in qPCR

| Gene | Forward primer sequence (5’-3’) | Reverse primer sequence (5’-3’) |
| --- | --- | --- |
| GAPDH | TGACTTCAACAGCGACACCCA | CACCCTGTTGCTGTAGCCAAA |
| PSMC2 | CAGCACTCTGGGATTTGGCT | TTTCTATCCACGCCCACTCTC |
| PLAU | GGGGAGATGAAGTTTGAGGTG | GCAATGTCGTTGTGGTGAGC |
| PLAC8 | CACATTTTGTTTCCCGTGCCT | TCATTGCGACGCTTGTTCC |
| BIRC5 | TCTCAAGGACCACCGCATCT | TTTGCATGGGGTCGTCATCT |
| KIF20A | GGCCGTTCCTGCATGATTGT | TGTCTGCCTTAGCCCCTTTCT |
| CCNB2 | AAGTTCCAGTTCAACCCACCA | GCAGAGCAAGGCATCAGAAAA |
| DDIT4 | TTAGCAGTTCTCGCTGACCG | CCAAAGGCTAGGCATGGTGA |
| CCND3 | TACCTGGATCGCTACCTGTCTT | GGTCGGTGTAGATGCACAGTTTT |
| CCND1 | AGCTGTGCATCTACACCGAC | GAAATCGTGCGGGGTCATTG |
| SAC3D1 | CAAAGCGGTTGGTTGCGG | CGCGCTCCCCTTAGTGTTTT |
| E2F8 | TGACGAAGTGGCAGAGGAAC | CATCATAATCTGCTCGGCGTA |
| CCNA2 | AGCCTGCGTTCACCATTCA | GGGCATCTTCACGCTCTATTTT |
| HAUS1 | GTGAATACGAGTCAGAAGCCAAGT | CACCGCACTGTCAACCAAA |
| CCNA1 | TGGAAAGAAAGCACTCCCTGAC | TCCTCAAATGCCATCCCCTC |
| CDCA8 | TTGAGTCAGACAGGCAGAACC | TTCCTCCAAGGGCGAAGTAG |
| CENPK | CAACGGTGGTTGGATGAACA | GAAACTCGCCCAAGGTACTCA |
| SSX2IP | AGCTTTACAGAAGCCGCTATTC | TGAGTTCTGGTGGTCAAAGGTAG |
| MAPK9 | CTCTGCGTCACCCATACATCA | TCTTTCTTCCAACTGGGCATC |
| SUSD3 | ACCTTTGGCTTCAAGGTGG | GCTTGCTCTTCTTCACGCACT |
| RDM1 | GCCCATCCTGGTTTCTATGC | GGCTTGATGTTGAACTGCCTTA |
| DEPDC1B | CTGAAGTGACCCGCAAACAAA | CTGGTGGGAGATCATTCCATTC |
