# Supplementary material for: Overexpression of PSMC2 promotes the tumorigenesis and development of human breast cancer via regulating plasminogen activator urokinase (PLAU)
Source: Cell Death Dis. 2021 Jul 9;12(7):690. doi: 10.1038/s41419-021-03960-w (PMC8271021; doi:10.1038/s41419-021-03960-w)
Supplement: Supplementary file 4 — Table S4 [file 41419_2021_3960_MOESM4_ESM.docx]

Table S4 Relationship between PSMC2 expression and tumor characteristics in patients with breast cancer analyzed by Spearman correlation analysis

| Tumor characteristics | index |  |
| --- | --- | --- |
| Grade | Spearman correlation | 0.227 |
|  | Significance (two tailed) | 0.005 |
|  | n | 149 |
| Tumor size | Spearman correlation | 0.193 |
|  | Significance (two tailed) | 0.020 |
|  | n | 146 |
