# Supplementary material for: Overexpression of PSMC2 promotes the tumorigenesis and development of human breast cancer via regulating plasminogen activator urokinase (PLAU)
Source: Cell Death Dis. 2021 Jul 9;12(7):690. doi: 10.1038/s41419-021-03960-w (PMC8271021; doi:10.1038/s41419-021-03960-w)
Supplement: Supplementary file 5 — Supplementary figure legends [file 41419_2021_3960_MOESM5_ESM.docx]

**Figure S1.** (A) The endogenous expression of PSMC2 in breast epithelial cell line HBL-100 and breast cancer cell lines including MCF-7 and MDA-MB-231 was detected by qPCR. Data were shown as mean ± SD (n ≥ 3). ** P < 0.01

**Figure S2.** (A) The transfection efficiencies of shPSMC2 and shCtrl, knockdown efficiency of PSMC2 in MCF-7 cells were evaluated by fluorescence imaging, qPCR and western blotting, respectively. (B) The transfection efficiencies of shPSMC2 and shCtrl, knockdown efficiency of PSMC2 in MDA-MB-231 cells were evaluated by fluorescence imaging, qPCR and western blotting, respectively. Data were shown as mean ± SD (n ≥ 3). *** P < 0.001

**Figure S3.** (A) A human apoptosis antibody array was used to detect the expression of apoptosis-related proteins in breast cancer cells with or without PSMC2 knockdown. (B) Based on the results of human apoptosis antibody array, several differentially expressed proteins in apoptosis-related signaling pathways were identified and shown. The data are shown as mean ± SD. * P < 0.05

**Figure S4.** The Ki67-positive cell ratio in xenografts collected from shCtrl or shPSMC2 group was evaluated.

**Figure S5.** (A) A volcano plot of gene expression profiles in MDA-MB-231 cells with or without PSMC2 knockdown. The green dots represent the downregulated DEGs. The red dots represent the upregulated DEGs. (B) The enrichment of the DEGs in canonical signaling pathways was analyzed by IPA. (C) The enrichment of the DEGs in IPA disease and function was analyzed by IPA. (D) After the transfection of shCtrl or shPSMC2, cells were subjected to western blot analysis or immunoprecipitation with anti-PLAU. Western blot analysis for ubiquitinated proteins and ubiquitinated PLAU was performed to show the effects of PSMC2 knockdown. The data are shown as mean ± SD (n ≥ 3). * P < 0.05

**Figure S6.** The transfection efficiencies of shCtrl, shPLAU, shPSMC2+shPLAU in MDA-MB-231 cells were evaluated through observing the GFP fluorescence of the lentivirus vector.

**Figure S7.** The mRNA and protein expression of PLAU and PSMC2 in shCtrl, shPLAU and shPSMC2+shPLAU groups of MDA-MB-231 cells was detected by qPCR (A, B) and western blotting (C), respectively. The data are shown as mean ± SD (n ≥ 3). ** P < 0.01, *** P < 0.001

**Figure S8.** The mRNA and protein expression of PLAU and PSMC2 in shCtrl+Vector, PLAU+shCtrl, shPSMC2+Vector and shPSMC2+PLAU groups of MDA-MB-231 cells was detected by qPCR (A, B) and western blotting (C), respectively. The data are shown as mean ± SD (n ≥ 3). * P < 0.05, ** P < 0.01, *** P < 0.001
