# Supplementary figures and images for: Overexpression of PSMC2 promotes the tumorigenesis and development of human breast cancer via regulating plasminogen activator urokinase (PLAU)
Source: Cell Death Dis. 2021 Jul 9;12(7):690. doi: 10.1038/s41419-021-03960-w (PMC8271021; doi:10.1038/s41419-021-03960-w)

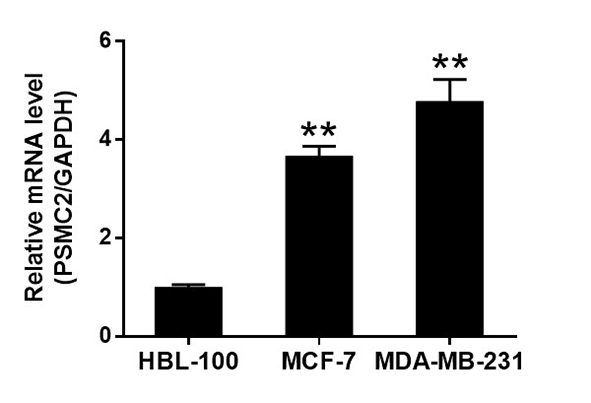

Supplement: Supplementary file 6 — Figure S1 [file 41419_2021_3960_MOESM6_ESM.tif]

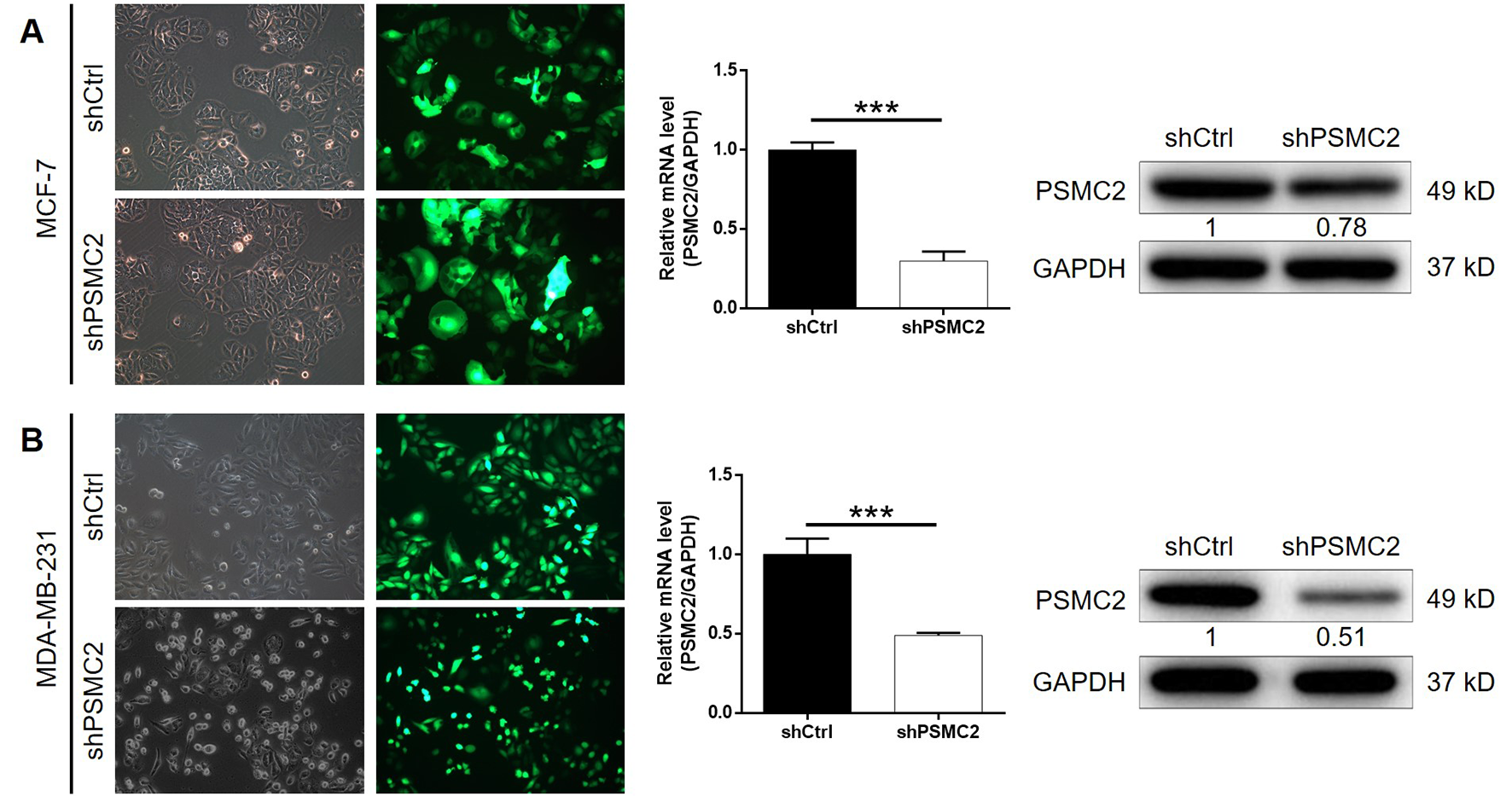

Supplement: Supplementary file 7 — Figure S2 [file 41419_2021_3960_MOESM7_ESM.tif]

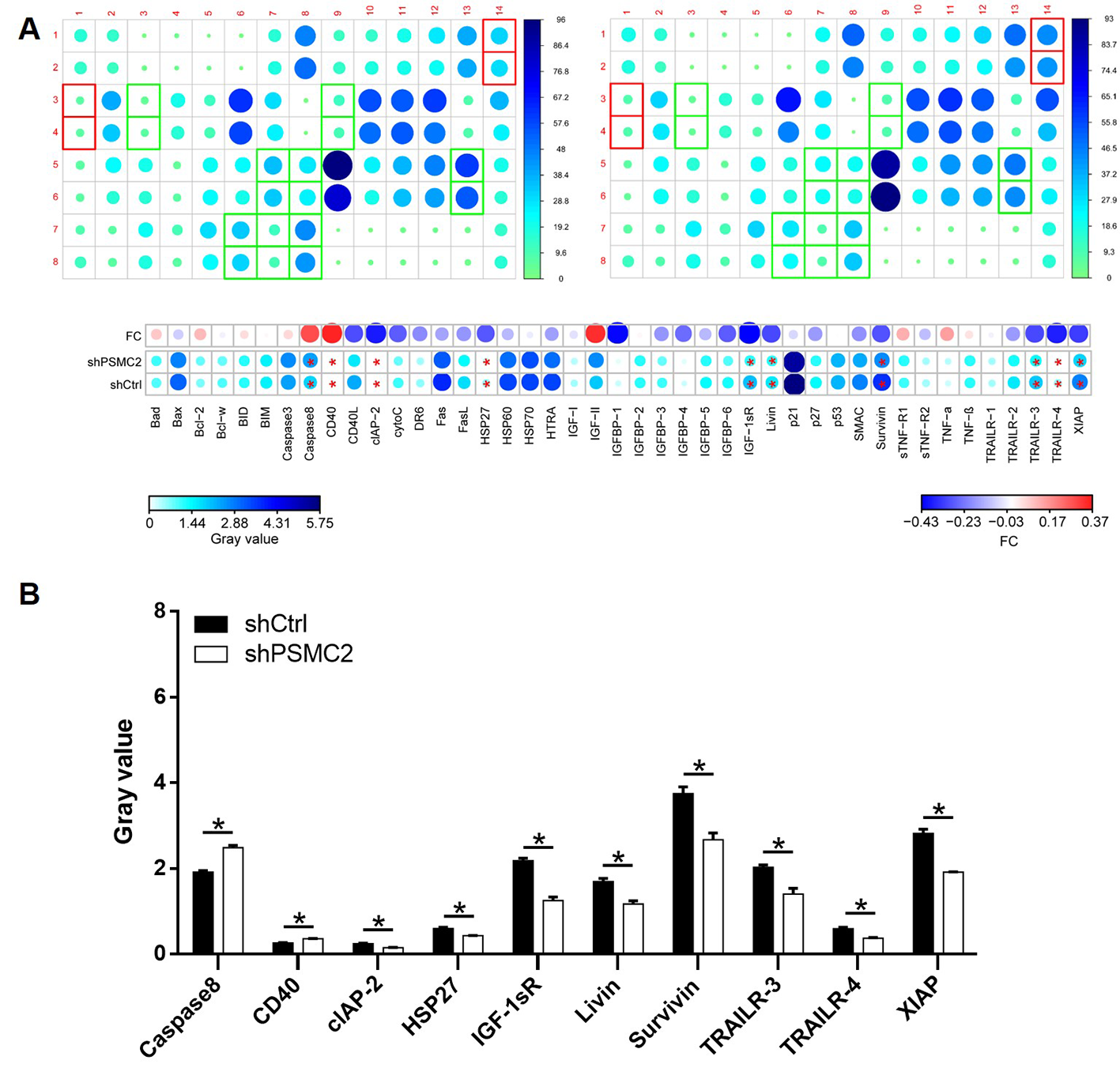

Supplement: Supplementary file 8 — Figure S3 [file 41419_2021_3960_MOESM8_ESM.tif]

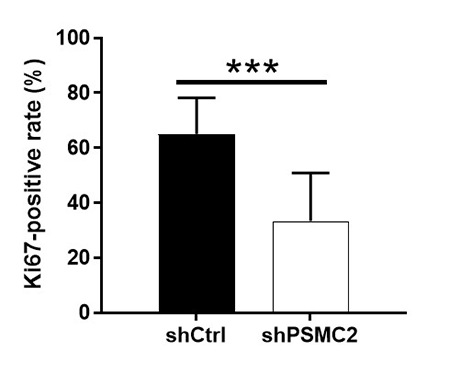

Supplement: Supplementary file 9 — Figure S4 [file 41419_2021_3960_MOESM9_ESM.tif]

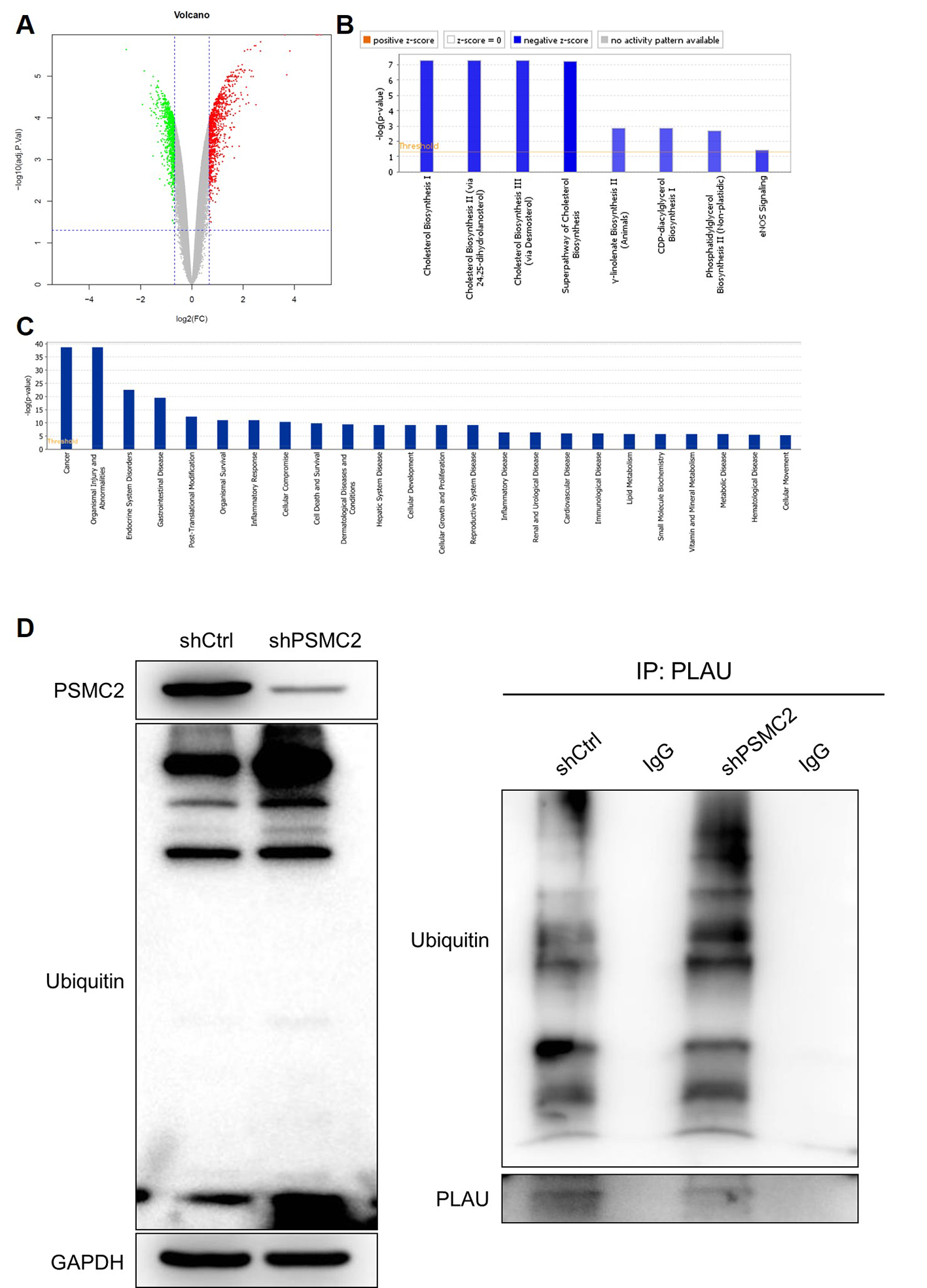

Supplement: Supplementary file 10 — Figure S5 [file 41419_2021_3960_MOESM10_ESM.tif]

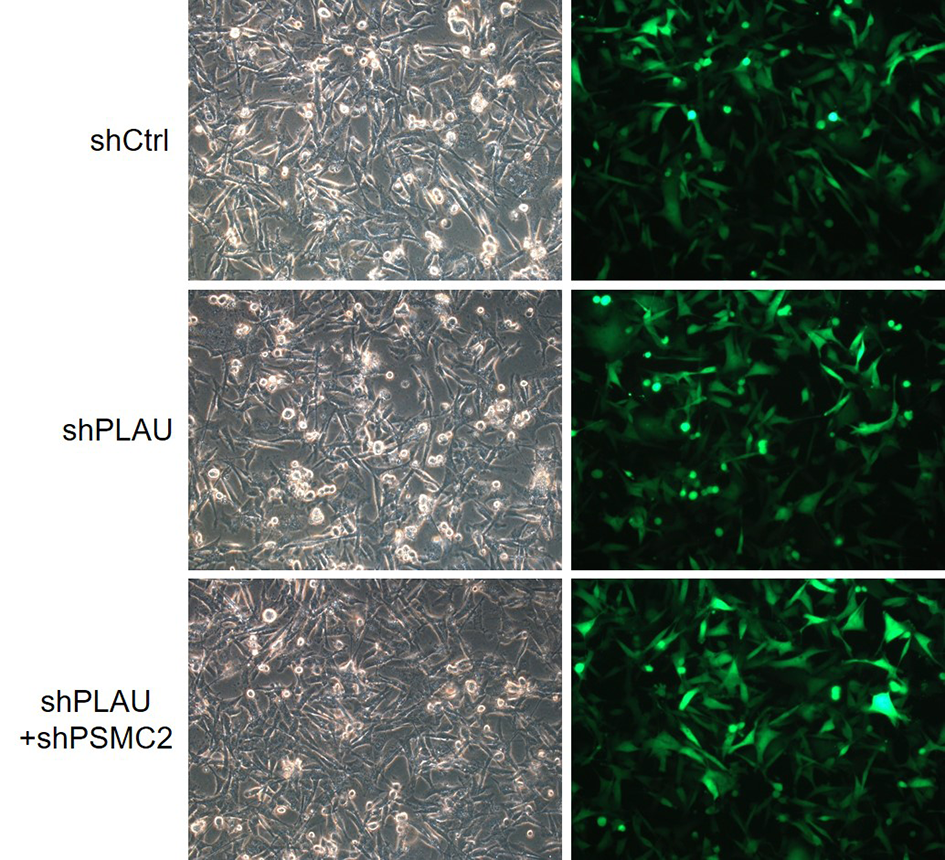

Supplement: Supplementary file 11 — Figure S6 [file 41419_2021_3960_MOESM11_ESM.tif]

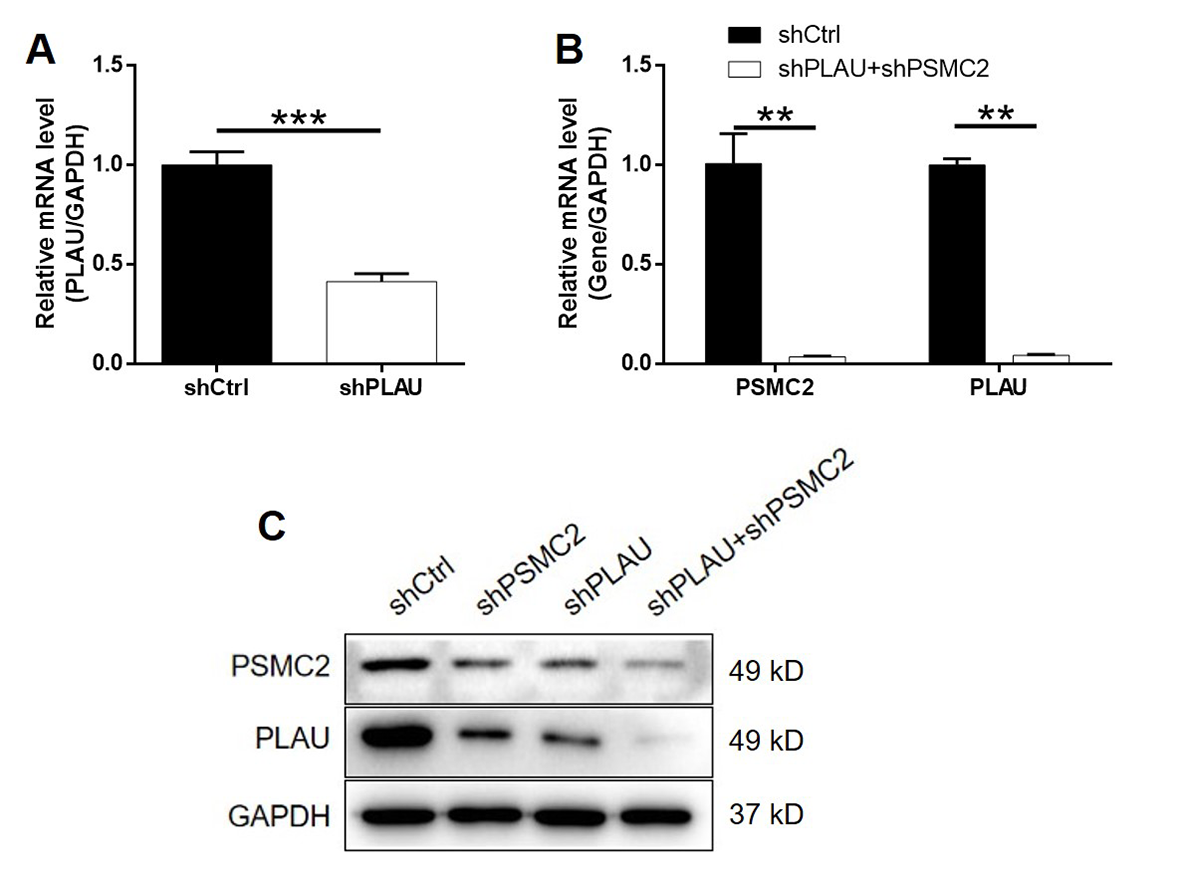

Supplement: Supplementary file 12 — Figure S7 [file 41419_2021_3960_MOESM12_ESM.tif]

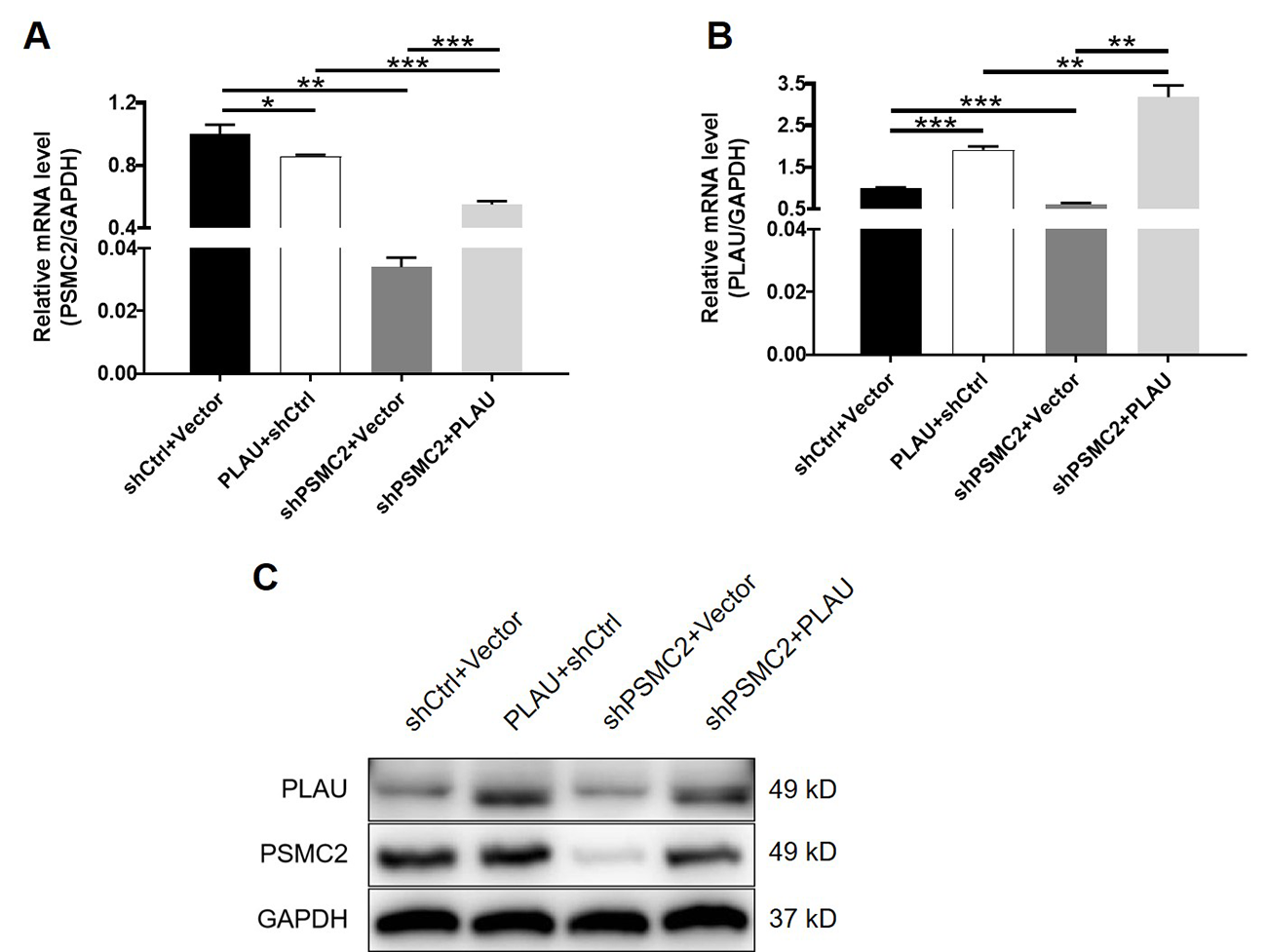

Supplement: Supplementary file 13 — Figure S8 [file 41419_2021_3960_MOESM13_ESM.tif]
